# UNDERSTANDING THE ADOPTION OF A MOBILE APPLICATION TO SUPPORT WORKFLOW OF HEALTHCARE AIDES

**DOI:** 10.1093/geroni/igac059.2194

**Published:** 2022-12-20

**Authors:** Christine Daum, Antonio Miguel-Cruz, Hector Perez, Emily Rutledge, Sharla King, Lili Liu

**Affiliations:** University of Waterloo, Waterloo, Ontario, Canada; University of Alberta, Edmonton, Alberta, Canada; University of Waterloo, Waterloo, Ontario, Canada; University of Waterloo, Waterloo, Ontario, Canada; University of Alberta, Edmonton, Alberta, Canada; University of Waterloo, Waterloo, Ontario, Canada

## Abstract

Healthcare aides are unlicensed support personnel who provide direct care, personal assistance and support to persons living with health conditions. Workflow issues have a negative impact on health care aides’ job satisfaction and quality of care. The implementation of information communication technologies could improve workflow. In collaboration with an industry partner, we developed a mobile application intended to support the workflow of health care aides who provide services to long-term care residents living with dementia. The purpose of this study was to investigate the technology acceptance and usability of a mobile application in a real-world environment when used by health care aides of a care facility. We used a sequential explanatory mixed methods approach. Our study included pre and post paper-based questionnaires with no control group (n=60). This was followed by two focus groups with a subsample of health care aides informed by qualitative description (n=12). We found: (a) acceptance of the mobile application was high; (b) usefulness was the strongest predictor of intention to use the mobile application, and (c) intention to use the mobile application predicted usage behaviour. Focus group findings supported the quantitative findings and highlighted participants’ strong belief that the mobile application was useful, portable, and reliable. An area for improvement was user interface adjustments. Overall, these results support the assertion that our mobile application assisted health care aides in addressing their workflow issues and thus, has potential to improve the quality of care provided.

